# Biomarkers in Inflammatory Myopathies—An Expanded Definition

**DOI:** 10.3389/fneur.2019.00554

**Published:** 2019-06-04

**Authors:** Olivier Benveniste, Hans-Hilmar Goebel, Werner Stenzel

**Affiliations:** ^1^Department of Internal Medicine and Clinical Immunology, Pitié-Salpêtrière University Hospital, Assistance Public-Hôpitaux de Paris, Sorbonne-Université, INSERM, UMR974, Paris, France; ^2^Department of Neuropathology, Berlin Institute of Health (BIH), Charité - Universitätsmedizin, corporate member of Freie Universität Berlin, Humboldt-Universität zu Berlin, Berlin, Germany; ^3^Department of Neuropathology, Mainz - Universitätsmedizin, Johannes Gutenberg- University, Mainz, Germany

**Keywords:** IIM, myositis-specific-autoantibodies, DM, IMNM, IBM, myositis, biomarker, morphology

## Abstract

Biomarkers as parameters of pathophysiological conditions can be of outmost relevance for inflammatory myopathies. They are particularly warranted to inform about diagnostic, prognostic, and therapeutic questions. As biomarkers become more and more relevant in daily routine, this review focusses on relevant aspects particularly addressing myopathological features. However, the level of evidence to use them in daily routine at presence is low, still since none of them has been validated in large cohorts of patients and rarely in independent biopsy series. Hence, they should be read as mere expert opinions. The evaluation of biomarkers as well as key biological parameters is an ongoing process, and we start learning about relevance of them, as we must recognize that pathophysiology of myositis is biologically incompletely understood. As such this approach should be considered an essay toward expansion of the definition “biomarker” to myositis, an emerging field of interest in biomedical research.

## Inflammatory Myopathies

Inflammatory myopathies may relate to different groups of diseases comprising infectious ones, those associated with other rheumatological or syndromic diseases affecting extramuscular systems and the ones, which occur as sole organ affection (muscle affection) in the context of a defined extramuscular disease. The group of inflammatory myopathies *sensu strictu* is termed the idiopathic inflammatory myopathies (IIMs) and they are again comprising heterogeneous entities (1–5).

For more than 40 years, the inflammatory myopathies (IIMs) have been assigned to either polymyositis (PM) or dermatomyositis (DM) (6, 7), and sporadic inclusion body myositis has also been included here. However, recently the spectrum of PM and DM has been rearranged, and this was achieved on the basis of the definition of subgroups with homogeneous clinical symptoms like e.g., the anti-synthetases syndrome and associated myositis (8–11). The sub-entities have also been confirmed at the serum auto-antibody level (12) and at the morphological level (2).

## Approved Definition of Biomarkers and Expanded Definition of Biomarkers

A biomarker is defined as an indicator of a certain physiological or pathophysiological condition. Biomarkers may also inform about prognosis and therapeutic effectiveness in times of targeted therapy approaches. They are warranted if a direct assessment of a condition or the function/dysfunction of an organ is not easily accessible. It may also be useful if time to render a firm diagnosis matters. Sensitivity and specificity are of outmost relevance if we talk about biomarkers and their interpretation. The National Institutes of Health (NIH) propose the following definition: A biomarker is: “a characteristic which is objectively measurable, indicating normal or pathophysiological processes, or treatment response to therapeutic intervention.” This implies two main items: (i) a biomarker should be measurable with precision and reliability. (ii) The potential indirect character of a biomarker based on one or several biological parameters (e.g., genetic characteristics, proteins, “key” molecules, metabolites, etc.), which allow characterization/description of a physiological or a pathological state, the evolution of a disease or its response to treatment. This may be called the *approved definition* of a biomarker.

In our daily practice, assessment of certain biomarkers is part of routine exams (e.g., blood sugar), whereas others are only assessed in very specific situations/diseases and measured in highly specialized laboratories. The whole field of laboratory medicine can be regarded as a biomarker repository for the individual human being and can be evaluated over time. Just to name some, in oncology we use enzymes (alkaline phosphatase) and also tumor proteins and more recently genetic alterations like *BRACA* to identify risk factors, activity of a cancer, or acquire information on prognosis and even on therapeutic decisions. The measurement of Dystrophin staining (intensity and expansion) is an interesting example of what we would like to call *expanded definition of biomarker use*. Dystrophin levels cannot be assessed in the serum or cerebrospinal fluid of patients to obtain information about the level of “left-over” dystrophin as a measure of therapeutic success of modern dystrophin replacement strategies.

Biomarkers we use in cardiology are Troponin to test cardiac injury or NT proBNP to test cardiac failure, both markers can be measured in the blood of patients. Levels of CD4^+^ cell count and HIV viral burden are used to monitor HIV treatment efficacy. Biomarkers we use in pulmonology are gasses like O_2_ and CO_2_. Biomarkers in forensic medicine may be blood alcohol and liver enzymes. In neurodegenerative diseases, certain CSF and blood parameters are indicative of disease activity, but it is difficult to gain information about thresholds and early stages of degenerative diseases.

If we take a look at chronic inflammatory diseases, we also use a number of interesting biomarkers that may inform about a certain entity: e.g., ANCAs in ANCA-associated vasculitis, and less specific markers such as ANA antinuclear antibodies, ENA, dsDNA etc., which just inform about connective tissue disorder classification or anti-DNA titer and/or complement dosages measuring disease activity in lupus erythematosus (13–15). In modern diagnostic approaches to autoimmune encephalitis, anti-neuronal antibodies like NMDA or LGI1 and CASPR2 (Anti-voltage gated potassium channel associated proteins) are measurable in the serum and can be used as diagnostic markers (e.g., in brain slice cultures of rodents) (16), because for obvious reasons, the brain is not accessible to a biopsy without considerable risk. Myasthenia gravis has highly specific biomarkers such as e.g., anti-AchR or anti-MUSK antibodies (17). However, in other chronic inflammatory CNS diseases like multiple sclerosis, unfortunately there is no widely-accepted highly specific marker in the serum. Instead, we generally use CSF markers like oligoclonal bands (OCBs) that are not present in serum, to achieve diagnostic certainty, although OCBs are not at all specific for multiple sclerosis.

## Biomarkers in Inflammatory Myopathies

If we want to define biomarkers we should ask for what they may be useful, hence if we need them for diagnostic or prognostic accuracy and clinical follow-up, or if we need them for therapeutic decisions (as well), as a biomarker “companion with medication.” The latter can be measured only once to establish a certain therapy or multiple times during therapy to monitor efficacy or toxicity.

### Which Biomarkers Can Be Used in Muscle Diseases?

Biomarkers in the narrower sense are considered to be measurable in bodily liquids, however, there may also be certain patterns: e.g., morphological patterns (2, 8, 18), or MRI-patterns. The latter have attracted great interest, specifically in congenital myopathies like core myopathies, and CMDs like Ullrich muscular dystrophy to a point that they can predict genetic mutations with high certainty (19).

## Muscle Enzymes and Related Molecules as Biomarkers

There are five “muscle enzymes” including creatine kinase (CK), transaminases: aspartate aminotransferase (AST) and alanine aminotransferase (ALT), lactate dehydrogenase (LDH), and aldolase, which leak into the circulation from damaged muscle leading to their elevation in serum. Moderate to high correlations were observed among them (20). All of them have been used as indirect markers of any condition inducing myolysis, including the idiopathic inflammatory myopathies (IIM). Some of these enzymes are more specific of muscle tissue (CK, aldolase), while others are present in nearly all living cells (LDH) or in hepatocytes (transaminases). One of the most common causes of CK elevation is eccentric exercise. Serum levels depend on gender, muscle mass, exercise intensity, and duration in addition to the individual training state, and there is a remarkable inter-individual variability in the degree to which serum enzyme activities increase with exercise (21, 22). Thus, one must first re-test these enzymes at rest, at least 5–7 days after physical activity or any eccentric exercise, as the peak of CK often occurs at 4 days delay (23). After excessively intense exercise, muscle enzyme release cannot be used to predict the magnitude of muscle function impairment caused by muscle necrosis (24). That is, CK levels up to 100,000 IU/L can be perfectly asymptomatic or reveal an exertional heat illness with rhabdomyolysis. Conversely, some skeletal muscle diseases (Myotonic dystrophy, congenital myotonia, neurogenic disorders and myasthenia) may not show elevated CK levels at all while the clinical impairment can be very considerable. Similar muscle enzyme leakage into the blood can be observed in many muscle diseases with muscle fiber necrosis from rhabdomyolysis (toxic, genetic, heat illness) to inherited dystrophies or metabolic myopathies or IIM, as well as during mechanical (25) or electrical (26) injuries.

Given these limitations, serum CK levels are generally good markers of disease activity in myositis. However, in certain forms of dermatomyositis (27) and inclusion body myositis (28) patients' CK levels can be slightly elevated or normal, completely independent of muscle weakness or disease severity; so, they are not suitable markers of disease activity in these conditions. In DM patients, notably those with anti-Mi-2 antibodies, CK levels appear elevated (often > 5,000 IU/L) at onset and normalize with treatment (Landon-Cardinal O. Anti-Mi2 Dermatomyositis Revisited: Pure DM Phenotype with Muscle Fiber Necrosis and High Risk of Malignancy. In: *ACR Meeting Abstracts*. Available at: http://acrabstracts.org/abstract/anti-mi2-dermatomyositis-revisited-pure-dm-phenotype-with-muscle-fiber-necrosis-and-high-risk-of-malignancy/. Accessed January 31, 2017) so following levels in individual patients is reasonable in Mi-2^+^ dermatomyositis. In patients with anti-Jo-1^+^ anti-synthetase syndrome (29), and immune mediated necrotizing myopathies with anti-SRP (30) or anti-HMGCR (31) antibodies, CK levels clearly correlate with myofiber necrosis and thus disease activity and should be used in the follow-up of the patients. CK levels obviously do not allow for differentiation between IIMs and other e.g., genetic/metabolic muscle diseases and they cannot be used to differentiate between different IIM subtypes, although some IIMs have tendency to show high CK levels than others (IMNM>ASSM>DM, OM, NM>IBM).

Several other laboratory markers which are generally assessed in routine blood exams can be used as biomarkers. Among those are KL-6, ferritin, and troponins: KL-6 has been shown to be useful biomarkers for monitoring activity and severity of ILD in DM and PM as well as in jDM (32, 33). Ferritin was analyzed as a biomarker with similar profile as KL-6 and correlates well with treatment responsivity, specifically in anti-MDA5-associated DM (34, 35). Troponins (serum Troponin T) were assessed in addition to CK and CK-MB ratio early on in PM and DM and are useful markers as well (36, 37) Also in sIBM the heart and the value of assessing troponins was tested but was not found different to an age-matched control group (38). TnT values were elevated in another study however not reflecting cardiac damage (39). All the cited serum biomarkers have mostly grade II level of evidence (max. smaller randomized control trials or series or case-control studies).

## The Interferon Signature as Biomarker

Transcriptomic studies carried out on biopsy specimens from skeletal muscle from DM patients have shown a specific up-regulation of multiple interferon-stimulated genes (ISG) suggesting that type I interferons (IFN-I) play an important role (40, 41). The expression of some interferon signature genes (ISGs), such as MXA, ISG15, and RIG-I, has been confirmed at the protein level in perifascicular regions and on the capillaries of the muscle biopsies (40–44). DM patients harbor high levels of circulating IFN-I cytokines including IFN-β (45) and IFN-α (46, 47), and the disease activity positively correlates with ISG transcript levels in the blood (48). In humans, there are five different types of type I interferons (IFN-I): IFN-α, IFN-β, IFN-ε, IFN-κ and IFN-ω (49). They are recognized by heterodimeric receptor complexes, comprising IFN-α receptor (IFNAR1) and IFNAR2 subunits that transduce signals to the nucleus by the JAK/STAT complex resulting in the upregulation of hundreds of different ISGs, including IFN-I cytokines, involved in anti-viral defense (50). While the IFN-I pathway has been implicated in the pathophysiology of DM for more than a decade, its role in muscle and skin damage has been precisely explored only recently (51). *In vitro*, the activation of IFN-I in differentiating myoblasts abolished myotube formation with reduced myogenin expression, while in differentiated myotubes, a reduction in surface area and an upregulation of atrophy-associated genes was observed. Still *in vitro*, exposure of endothelial cells to IFN-I disrupted vascular network organization. All the pathogenic effects observed *in vitro* were abolished by ruxolitinib (a JAK/STAT inhibitor) (51). Finally, *in vivo*, some refractory DM patients (in our hands today 10, of whom 4 have been reported in Ladislau et al. (51) were treated with ruxolitinib, and improvement ensued in skin lesions, muscle weakness and reduced serum IFN-I levels and interferon-inducible genes scores. Apparently, Janus kinase (JAK) inhibition is a promising mechanism-based treatment for DM, where IFN-I evaluation (52) (either in the serum and/or in the biopsy) might be a good biomarker for decision-making (51) (Table 2).

## Autoantibodies as Biomarkers

**Figure 1:**
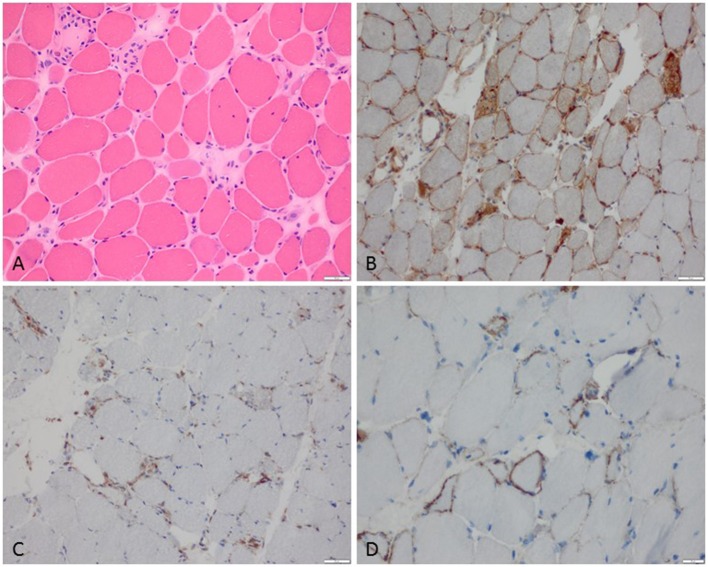
Characteristic example of anti-SRP+ IMNM. **(A)** Diffuse myofiber necrosis in different stages of single cell necrosis and regeneration (H&E stain, original magnification x200). **(B)** MHC class I sarcolemmal stain with diffuse character (original magnification x200). **(C)** CD68+ macrophages confined to myophagocytosis and diffusely distributed in the endomysium (original magnification x200). **(D)** C5b-9 complement deposition on the sarcolemma of myofibers (original magnification x400).

**Figure 2:**
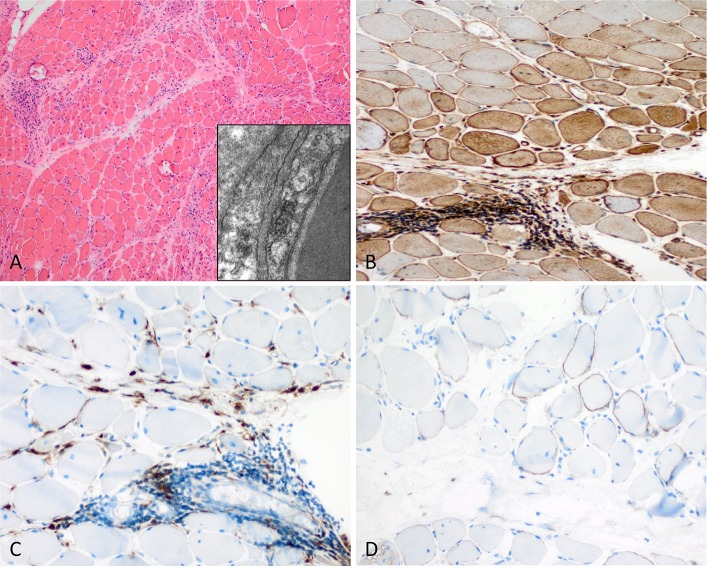
Characteristic example of anti-Mi2+ DM. **(A)** Perifascicular atrophy of myofibers (PFA) (H&E stain, original magnification x100). Electron microscopy: endothelial tubuloreticular inclusions in endothelial cells (original magnification x30.000). **(B)** Perifascicular MHC class I staining with a decreasing gradient toward the centrofascicular region (original magnification x200). **(C)** Perimysial macrophage infiltrate with extension to the endomysium (CD68, original magnification x200). **(D)** C5b-9 complement on the sarcolemma of myofibers (original magnification x200).

**Figure 3:**
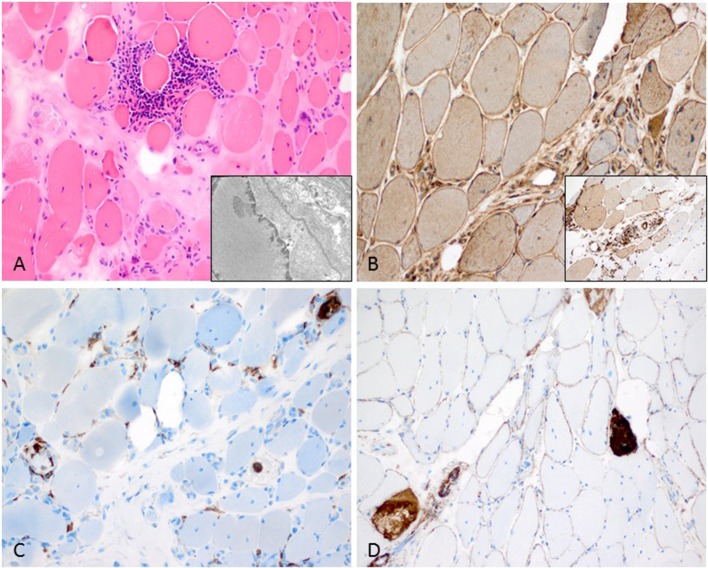
Characteristic anti-Jo1-positive ASS-associated myositis. **(A)** Necrotic myofibers confined to the perifascicular region (H&E stain, original magnification x200). Electron microscopy: intranuclear actin inclusions in myonuclei (insert; original magnification x20.000). **(B)** Sarcolemmal MHC class I stain is diffusely positive (original magnification x200) and MHC class II confined to the sarcolemma and sarcoplams of the perimysial myofibers (insert; original magnification x200). **(C)** Lympho-monocytic infiltrate extends into the endomysium (CD68+ macrophages and lymphocytes (original magnification x200). **(D)** Sarcolemmal C5b-9 and necrotic myofibers predominant in the perifascicular region (original magnification x200).

**Figure 4:**
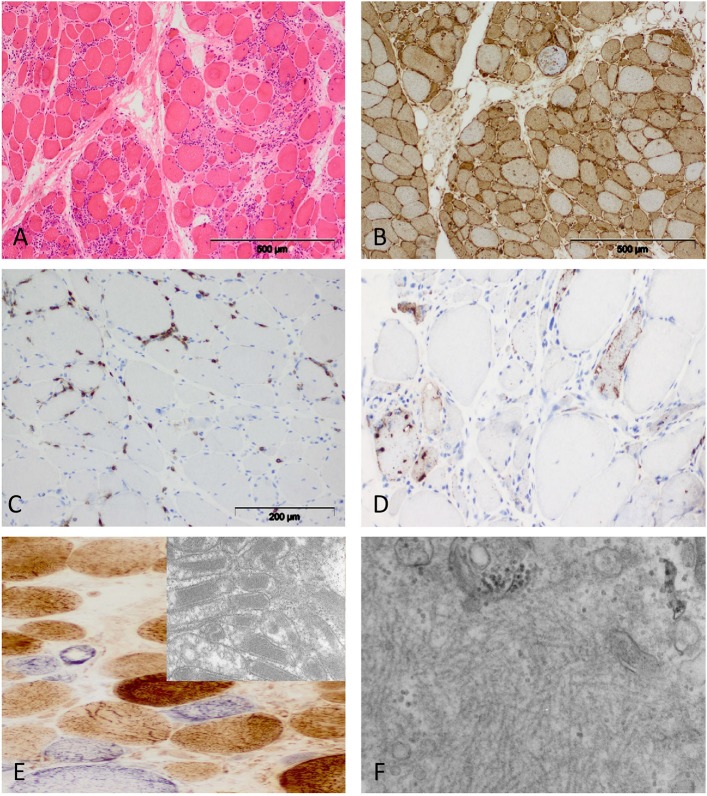
Characteristic morphology of sIBM. **(A)** Diffusely distributed necrotic myofibers in a severely myopathic tissue (original magnification x100). **(B)** Strong sarcolemmal and sarcoplasmic MHC class I staining (MHC class II fokal stain but no perifascicular pattern) [original magnification x100 (not shown)]. **(C)** Dense endomysial lymphocytic infiltrate (original magnification x200). **(D)** Presence of e.g., p62+ vacuoles in the sarcoplasm (original magnification x200). **(E)** Mitochondrial pathology with many COX-negative and SDH-positive fibers (original magnification x400) and paracristalline inclusions on EM (original magnification x20.000). **(F)** Electron microscopy: tubulofilaments (original magnification x30.000).

**Figure 5:**
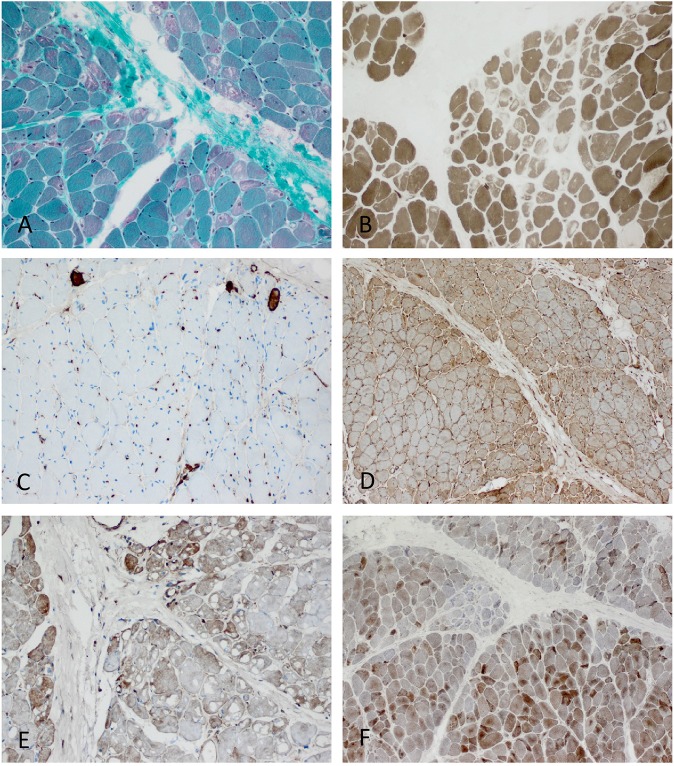
Characteristic example of anti-TIF1γ+ DM. Perifascicular pathology of myofibers (PFP) with: **(A)** atrophic fibers, punched-out vacuoles and violaceous fibers on Gömöri trichrome (original magnification x100). **(B)** abundant ghost fibers at the edge of fascicles (original magnification x100). **(C)** predominant complement (C5b-9) deposits on capillaries (original magnification x100). **(D)** MHC class I staining with perifascicular to centrofascicular gradient (original magnification x100). **(E)** MxA stain highlighting interferon signature-related pathology predominantly in the perifascicular region (original magnification x100). **(F)** Presence of COX paleness in the perifascicular region (original magnification x100).

A comprehensive number of autoantibodies have been identified both in childhood and in adults IIM: 5 for DM, 8 for ASS-associated myositis, 2 MSAs for IMNM, and cN1A for IBM (reviewed in this issue by Feist et al.). It is now well-established that certain MSAs identify typical clinically homogeneous subgroups of myositis (1, 2, 12, 53, 54) (Tables 1, 2 and Figures 1–5). Nevertheless, there may be variability in clinical severity, and also due to ethnical differences and hence underlying possible disease susceptibility genes, which may have an influence on the individuals' immune system), but this has not extensively been explored in IIMs yet (55).

**Table 1:** Morphological and combined morphological patterns increasing diagnostic accuracy and precision.

		**Morphological** **pattern** **(predominant)**	**autoantibody**	**Morphological** **diagnosis**
	**Conventional histology**	PFP-DM(+++ – +/–)	Mi2, TIF1γ, NXP2, SAE, MDA5	DM
		PFP-ASS(+++ – +)	Jo1, PL7, PL12, OJ, EJ etc.	ASSM
		Diffuse scattered myofiber necrosis & regeneration	SRP HMGCR	IMNM
		Necrosis diffuse and focally scattered	n/a	iRMyositis
		Necrosis and granuloma-like inflammation	M2	Anti-M2-associated Myositis
		Dystrophy-like pattern with rimmed vacuoles and inflammation	cN1a	sIBM severe
		Dystrophy-like pattern with rimmed vacuoles and inflammation	n/a	sIBM
		Granuloma in perimysium, perivascular or endomysium	n/a	Muscular sarcoidosis GvHD Myasthenia gravis (exceptional) etc.
**COMBINED PATTERNS**				
	PFP focal & focal necrotic fibers	MAC predominant on sarcolemma		Mi-2
	PFP +++	MAC predominant on capillaries		NXP2 or TIF1γ
PFP+ MHC I +++	MAC predominant on capillaries	Ghost fibers and punched-out vacuoles	Few T cells, many endomysial macrophages	Cancer associated TIF1γ DM
PFP+ MHC I +++	No/sparse MAC on capillaries	No or few ghost fibers and sparse punched-out vacuoles	Few T cells, many endomysial macrophages	At time of biopsy No cancer associated TIF1γ DM
PFP+ MHC I ++	Regional myofiber necrosis possible	MAC on capillaries and sarcolemma	T cells and few B cells, Many endomysial macrophages	NXP2-associated DM
PFP focal necrotic fibers focal MHC I ++	Occasional focal necrotic myofibers	MAC on sarcolemma AP may be positive in perimysium	B cells and T cells in perimysium and perivascular, macrophages	Mi-2-associated DM
PFP minor and focal MHC I +	No necrotic fibers	Occasional sarcolemmal MAC	Only sparse and focal infiltrate	MDA5-associated DM
No PFP-DM no PFP-ASS MHC I +++ diffuse and MHC II focal	Diffuse myofiber necrosis and fibrosis (dystrophy-like)	Rimmed vacuoles	Mitochondrial pathology	sIBM
PFP MHC I +++ MHC II ++	Perifascicular necrotic fibers or diffuse myofiber necrosis	MAC on sarcolemma	T cells and few B cells	Overlap Myositis with MAAs like anti-KU, -U1RNP, etc.

*PFP-DM Perifascicular pathology characteristic for DM*:

*This is the core feature, which is unifying all subtypes of DM while distinguishing them from other IIMs: It can be identified by a combination of stains which highlight the physiological effect of the Interferon type I-related pathology, loss of capillaries, atrophy of myofibers, fibers most often clustering in the perifascicular region during the course of disease, Non-specific stains that can be used to highlight this pathology are neo Myosin heavy chain (MyHc), MHC class I, CD56, complement (C5b-9), utrophin, laminin alpha5 showing a characteristic gradient: perifascicular toward the centrofascicular region. Sarcolemmal stains such as dystroglycans and laminin aloha 5 also show the sarcolemmal integrity of by far most atrophic fibers. This feature may help to distinguish atrophic from necrotic fibers. Specific stains showing involvement of characteristic type I interferon-related pathology that should be used are MxA, ISG15, RIG1 etc. highlighting, in most cases, a gradient as well, while sometimes staining may be more diffusely positive. EM highlights tubuloreticular inclusions in endothelial cells and lymphocytes (level II evidence) (1–3)*.

PFP-ASS Perifascicular pathology characteristic for ASSM:

*This is the core feature unifying all ASSM subtypes of which the most frequent ones are anti-JO1-associated myositis followed by anti-PL7, -PL12, -OJ, and rarely the remaining four known ones. It can be identified by variably intense presence of necrotic myofibers confined to the perifascicular area and absence of necrotic fibers at the center of fascicles, and absence of clusters of necrotic fibers or regional necrosis. Of note, there is absence of MxA staining of the perifascicular fibers. EM may highlight pathognomonic nuclear actin inclusions. (level II evidence) (4–8)*.

**Table 2:** Non-specific and disease specific biomarkers in myositis.

	**Laboratory Biomarker**		**Aim**
Non-Disease specific	CK, AST, ALT, LDH, and aldolase, troponins, ferritin, KL6, leukocytes, lymphocytes etc.		To differentiate the stage of a disease, evolution, effect of therapy(?) and pathophysiology
	MAAs		Helps to differentiate the severity of disease or inform about overlap features
Disease specific	MSAs		Helps to diagnose the subentity of IIMs
	TIF1γ and MAC on capillaries		TIF1γ-associated adult DM cancer is highly likely to ensue or be present
	TIF1γ but no MAC on capillaries		TIF1γ-associated adult DM cancer is less likely
	cN1A	sIBM	Marker of severity Useful for diagnosis if clinical features or biopsy features are non-conclusive or atypical
	Janus Kinase (Jak) Type I IFN signature	DM	Helpful for diagnostic purposes In the future may be helpful for selection of candidate medication, which is likely to prove efficacy
	Type I IFN signature	(j)DM	Helpful for diagnostic purposes To identify or select individual patients who benefit from best risk/benefit ratio of certain therapies
	ASS-associated ABs	ASSM	No elevated cancer risk
	Anti-SRP	IMNM	No elevated cancer risk
	Anti-HMGCR	IMNM	20–30% cancer
	No detectable AB	IMNM	30% cancer

Other autoantibodies falling into the group of MAAs have been associated with certain disease courses, and pathological presentations e.g. anti-mitochondrial M2 antibodies in granulomatous diseases (56) and necrotizing myopathy (Tables 1,2). However most of them like anti-PmSCL or anti-SSa or SSb and U1RNP have been described regularly in certain diseases like sclerodermia, Sjögren Syndrome, mixed connective tissue diseases etc., and we hypothesize that myositis may occur during these diseases rather than the antibodies occurring with myositis. Nevertheless, these associations may be very useful in terms of understanding of the pathogenicity of the autoantibodies since different “systems” such as muscle, skin, fibrous tissue, joints, epithelial cells etc. may all have a common antigenic target, a hypothesis which has not yet been explored in more detail.

## Certain *Patterns* of Histologic Abnormalities (Biomarkers From a Morphological Point of View)

Patterns of histological abnormalities can be very useful for diagnosis and are used in daily routine in myopathology. In general, our brain seems to function well in terms of pattern recognition and a pathologist's “eye” (& brain) is largely dependent on pattern recognition and comparison with certain standards/normals. However, a pattern has to be well-defined and there may be uncertainty or different definitions among diagnostic authorities. To unify concepts, it is of high importance to establish consensus internationally and also to critically question certain definitions (57–59).

Probably the most well-known morphological “biomarker” in this respect is the pattern of “perifascicular atrophy” (PFA), which is used to describe atrophic myofibers in the perifascicular region (the outer layers of a muscle fascicle in comparison to the less affected centrofascicular region). Of note, this atrophy may have various explanations in terms of pathophysiology and a small fiber may be purely atrophic but also represent a fiber in regeneration. Fiber atrophy certainly must not be confounded with fiber necrosis, although regeneration occurs as a consequence of necrosis and the cause of smallness of a single regenerating fiber may thus not be identifiable without having a look at other associated or consecutive features. PFA is the prime diagnostic feature of dermatomyositis although some entities may not show PFA so obviously (60) as others, and PFA is a time-sensible feature, which occurs only after some time and during progression of the disease. PFA may not overtly be apparent yet, hence several measures can be taken to document perifascicular pathology (PFP), which may not only be a less controversial nomenclature but also has the advantage that newer pathophysiological processes can be implemented (such as Cox paleness informing about mitochondrial pathology, and MxA stain informing about type I interferon-related pathology) (Figure 5). PFA can be highlighted by more classical immunohistochemical stains such as MHC class I stain (showing a decreasing gradient of staining intensity toward the center of the fascicles, which can be difficult to see in small fascicles). Another useful measure is to stain for CD56 and neonatal Myosin heavy chain (nMyHc) to ascertain affection of the perifascicular region (Figures 2, 5). In addition, and association with assessment of PFA, established pathophysiological concepts of DM such as increased ISGs (see above), can be used to highlight perifascicular pathology such as stains against MxA or ISG15, which may even be more sensible to identify DM-specific (perifascicular) pathology (44) than established MHC class 1 (61).

Another useful pattern in terms of perifascicular pathology may be termed perifascicular necrosis (PFN) highlighting necrotic muscle fibers predominantly in the perifascicular region (again compared to the centrofascicular region, which is not or much less affected). This pattern is characteristic of affected skeletal muscle in anti-synthetase syndrome (ASS)-associated myopathies such as those associated with antibodies directed against Jo1, PL7, PL12, OJ etc. (8–10) (Figure 3). PFN is not a characteristic feature of DM. In addition to this, MHC class I is strongly upregulated and can show a perifascicular gradient similar to DM, however, in case of doubt a helpful stain is MHC class II, which is strongly present in ASS-associated myositis and not or only very weakly in DM (8–10, 62). Complement (C5b-9) staining is widely used in assessment of IIMs and can stain the sarcolemma and the capillaries. It is positive on perifascicular muscle fibers sarcolemmally in DM and ASS-associated myositis (ASSM), hence not allowing any differentiation between these entities, but it is not positive on capillaries in ASSM. MxA is constantly absent in ASSM (52). If complement is identified on the sarcolemma in the perifascicular region in a patient with DM-typical PFP, diagnosis will be anti-Mi2^+^ DM. If predominant complement deposition is identified on the capillaries it will be anti-TIF1γ^+^ DM, or more rarely anti-NXP2^+^ DM (in adult patients) (Tables 1–3). Complement (C5b-9) deposition on vessels in association with TIF1γ can be used as a sensitive prognostic marker of malignancy. This is an example of combination of several biological parameters increasing cancer prediction (TIF1γ only 70%, vs. complement on capillaries + punched-out vacuoles and TIF1γ–positivity 90%) (63) (Table 1 and Figure 5).

**Table 3:** Diagnostic and prognostic utility of biomarkers in myositis.

**Biomarker** **(from blood)**	**Diagnosis**	**Distinguish between** **subgroups**	**Disease** **management**	**Prediction** **of prognosis**
CK	×	–	×	+/–
Troponin	×	–	×	+/–
KL-6	×	–	×	+/–
IFN signature (serum)	×	×	×	?
IFN signature biopsy	×	×	×	?
Autoantibodies MSA	×	×	×	×
Autoantibodies MAA	×	–	×	×
**LEVEL OF EVIDENCE: GRADE II**				
**Biomarker** **(from skeletal muscle)**	**Diagnosis**	**Distinguish between** **subgroups**	**Disease** **management**	**Prediction** **of prognosis**
PFP^***^	×	–	×	+/–
Degree of Inflammation^**^	×	–	–	–
Distribution of Inflammation^**^	×	–	–	–
Distribution of necrotic myofibers^**^	×	×	–	–
Complement deposits on capillaries^**^	×	×	× (if considered with TIF1y in adults >40)	× (if considered with TIF1y in adults >40)
Pattern MHC cl I^**^	×	×	–	–
Pattern MHC cl II^**^	×	×	–	–
P62/LC3^***^	–	–	–	–
IFN signature Biopsy^**^	×	×	×	?
Endothelial Tubuloreticular Inclusions^**^	+	–	–	–
Nuclear actin filaments^**^	+	+	–	–
Tubulofilaments^**^	+	+	+	+

Level of evidence grade II ** or III***.

Marker molecules of type I Interferon can be used for staining procedures as well (40, 41, 43, 64). They are strongly positive in all forms of DM but not or only minimally staining structures within the skeletal muscle in other IIMs (61), hence these stains can be effectively used to highlight that ASSM does not fall into the category of DM and must be regarded as a separate entity (65). Ultrastructural features can be very useful in diagnosis of IIMs: Tubuloreticular inclusions (TRIs) in endothelial cells are an early sign of dermatomyositis (66) and most DM biopsies irrespective of the associated autoantibody, show TRIs, with the exception of anti-MDA5 DM which shows TRIs in only 50% (60). TRIs are not specific, but highly sensitive for DM diagnosis. They can occur in some ASSM and SLE as well as HIV-associated myopathies, and in rare cases of sIBM TRIs have been noticed. Again, this is an example of the importance to combine certain biological parameters increasing their diagnostic accuracy. Myonuclear actin aggregates have so far only been found in ASS-associated myositis, mainly in anti-Jo-1^+^ patients (8) (Figure 3A, insert). Tubulofilaments associated with vacuoles and/or in myonuclei are characteristic biological parameters for sIBM and they are highly specific (Figure 4F), however their presence is not necessarily required for diagnosis (67). The authors' personal conviction is that these ultrastructural abnormalities have to be searched for thoroughly, and this may require time and expert knowledge, hence they are very useful if found.

The diagnosis of sIBM can be made based on clinical parameters/symptoms: The combination of hip flexor paresis and subsequent finger flexor paresis associated or not with swallowing difficulties in an elderly patient is very assuring (28). Light microscopical biological parameters are the combination of rimmed vacuoles, severe inflammation, mitochondrial abnormalities in a severely myopathic “dystrophy-like” biopsy (fiber necrosis, endomysial fibrosis and severe fiber-size variation). Additional biological staining parameters are p62, LC3, desmin, TDP43, and others—however their mere “presence” is not specific *per se*, they must show a focal coarse pattern (68, 69) and their presence in the context of the above-mentioned clinical picture can be used as a biomarker informing about a pathophysiological process relevant in this specific disease (Figure 4). However, congophilic inclusions within myofibers do not inform about beta amyloid deposits! It is a widely spread misconception that presence of congophilic material is equivalent to presence of beta amyloid! On the other hand, a convincing immunoelectron microscopic study has shown occasional beta- amyloid in myofibers (70), but this is not the bulk of amyloidogenic proteins, which may be present in sIBM (47). Moreover, current proteomic studies have shown that a multitude of different proteins can be found in vacuoles of sIBM biopsies, some of which are probably informative about certain interesting genetic backgrounds such as FYVE and coiled-coil domain containing 1 (FYCO1) (47) or valosin containing protein (VCP) (71).

The role of the autoantibody cN1A has been studied by different groups, and its clinical diagnostic use as a biomarker for sIBM is now accepted. Presence of the antibody in sIBM informs about severity of the disease course. However, the antibody has also been found outside of the context of myopathies in systemic sclerosis and systemic lupus (71).

## Future Aspects of Morphological Analysis in Routine and Research

To date, a certain panel of diagnostic stains should be performed by every myopathologist who reads muscle biopsies of myositis patients (58). In addition, EM should be performed in certain cases to increase diagnostic accuracy. New patterns of ultrastructural analysis such as the myonuclear actin inclusions may become apparent as we study more biopsies (8).

Combined immunohistochemical or double stains can inform about certain pathomechanisms linking them to each other and implementing newer pathophysiological concepts. It has to be defined if this approach is useful and necessary in routine diagnosis or if this can be used as biological parameters in research.

Proteomic approaches can help to identify and define molecules that are relevant to be studied in more depth and hence have the potential to become a biomarker for diagnosis, treatment and/or prognosis (47). Inflammatory patterns with CD8^+^ T cells, which surround and/or invade non-necrotic myofibers have been used traditionally as a diagnostic marker for IBM and PM. However, this feature is not at all specific and can be found in numerous monogenic diseases such as Facioscapulohumeral muscular dystrophy (FSHD), dysferlinopathy, anoctaminopathy, in lipid storage myopathies like multiple acyl-coenzyme A dehydrogenase deficiency (MADD), in toxic myopathies etc. (57). Other patterns as presence of B cells in clusters or in follicles can be highly suggestive of DM, and are associated with unfavorable outcome in jDM (72). B-cell follicles or accumulations can typically occur in a rare disease called brachiofacial myositis (73), and hence their presence in the context of the typical clinical picture can be defined as a biomarker to secure diagnosis of this rare and less well-known entity.

At presence, we would suggest to consider two of the above-mentioned biological parameters as relevant and very likely to be implemented in daily routine:

1. TIF1γ^+^ dermatomyositis in adults above the age of 40 years, with a skeletal muscle biopsy showing strong complement deposition on capillaries, punched-out vacuoles and ghost fibers can be considered to be highly suggestive of having or developing a cancer in the course of disease [cancer associated myositis likeliness between 50 and 90%; 84% in (63)].
2. A characteristic type I interferon signature that can be highlighted in the skeletal muscle tissue [at present most easily and reliably identified by staining for MxA (44, 61)].
3. Characteristic morphological phenotypes on muscle biopsies, which inform about the precise diagnosis of myositis subentities, best to be used in combination with clinical and auto-antibody information (this ‘biomarker’ is probably better called a ‘set of diagnostic features’, which however is essential to provide the most precise diagnosis we can provide for patients who may likely need an individualized therapeutic scheme).

In the future, successful therapeutic interventions may be used as biomarkers and secure diagnosis in rare unclear cases as well. Conversely, we may identify biomarkers informative about therapeutic success in IIMs as well.

## Author Contributions

WS, H-HG, and OB all have drafted, written, and corrected the paper together.

### Conflict of Interest Statement

The authors declare that the research was conducted in the absence of any commercial or financial relationships that could be construed as a potential conflict of interest.

## Abbreviations

PFP: perifascicular pathology
ASS: Anti-synthetases syndrome
ASSM: Anti-synthetases syndrome-associated myositis
MxA: Myxovirus A
EM: Electron microscopy
ISG15: Interferon-stimulated gene 15
RIG1: retinoic acid inducible gene I
CD56 NCAM: Neural cell adhesion molecule
MAC: C5b-9 Membrane attack complex
IIMs: Idiopathic inflammatory myopathies
MAA: Myositis-associated autoantibodies
MSA: Myositis-specific autoantibodies
Anti-M2: anti mitochondrial Antibodies
DM: dermatomyositis
IMNM: Immune-mediated necrotizing myopathy
iRMyositis: immune checkpoint inhibitor-related myositis
Anti-M2-asociated myositis: Anti Mitochondrial antibody-associated myositis
sIBM: sporadic inclusion body myositis
GVHD: graft vs. host disease
AP: alkaline phosphatase
MHC-class I (-II): major histocompatibility complex.
